# The risk factors of neuropathic pain in neuromyelitis optica spectrum disorder: a retrospective case-cohort study

**DOI:** 10.1186/s12883-022-02841-9

**Published:** 2022-08-19

**Authors:** Xiaojun Li, Haoyou Xu, Zequan Zheng, Huiying Ouyang, Guixian Chen, Zhenzhen Lou, Haoxuan Chen, Jiahui Zhang, Yibo Zhan, Hui Mao, Changlin Zhang, Min Zhao, Yuanqi Zhao

**Affiliations:** 1 The Second Clinical College of Guangzhou, University of Chinese Medicine, Guangzhou, 510006 China; 2 Department of Neurology, Guangdong Provincial Hospital of Chinese Medicine, The Second Affiliated Hospital of Guangzhou University of Chinese Medicine, 111 Dade Road, Guangzhou, 510120 China

**Keywords:** Neuromyelitis optica spectrum disorder, Neuropathic pain, AQP4-IgG antibody

## Abstract

**Background:**

Neuropathic pain is a common complication in neuromyelitis optica spectrum disorder (NMOSD), which seriously affects the quality of life of NMOSD patients, with no satisfactory treatment. And risk factors of neuropathic pain are still uncertain.

**Objective:**

To investigate the risk factors of neuropathic pain in a NMOSD cohort.

**Materials and methods:**

Our study was a retrospective case-cohort study, the patients diagnosed with NMOSD in the Department of Neurology from the Second Affiliated Hospital of Guangzhou University of Chinese Medicine from January 2011 to October 2021 were screened. Inclusion criteria were: (1) patients diagnosed as NMOSD according to the International Panel for NMO Diagnosis (IPND) criteria, (2) the aquaporin-4 immunoglobulin G antibodies (AQP4-IgG) test was performed. Patients without AQP4-IgG antibody were excluded. Clinical data, including sex, age of the first onset, symptoms of the first episode including neuropathic pain and attack types, localization of lesions of the first episode on Magnetic Resonance Imaging (MRI), Extended disability status Scale (EDSS) of the first onset, treatment of immunosuppression in the first acute phase, disease modifying therapy (DMT), treatment of neuropathic pain and APQ4-IgG status were collected from the hospital system database. Neuropathic pain was defined according to the International Association for the Study of Pain criteria and was described as “pain arising as a direct consequence of a lesion or disease affecting the somatosensory system”.

**Results:**

One hundred nineteen patients were screened and finally 86 patients fulfilling the inclusion and exclusion criteria were enrolled in our study. The prevalence of neuropathic pain in patients with NMOSD was 43.0%. Univariate analysis showed that the factors associated with neuropathic pain were the age at the onset, the attack type of optic neuritis, the attack type of myelitis, length of spinal cord involvement, localization of thoracic lesion, optic lesion, upper thoracic lesions, lower thoracic lesions, extended spinal cord lesions (≥ 3 spinal lesions), extended thoracic lesions (≥ 4 thoracic lesions), intravenous immunoglobulin and mycophenolate mofetil. Multivariate regression analysis showed that extended thoracic lesions (OR 20.21 [1.18–346.05], *P* = 0.038) and age (OR 1.35 (1–1.81) *P* = 0.050) were independently associated with neuropathic pain among NMOSD patients and that gender (OR 12.11 (0.97–151.64) *P* = 0.053) might be associated with neuropathic pain among NMOSD patients.

**Conclusion:**

Extended thoracic lesions (≥ 4 thoracic lesions), age and gender might be independent risk factors of neuropathic pain among patients with NMOSD. However, with a small sample size and predominantly female, caution must be applied and these results need validating in further cohorts.

## Introduction

Neuromyelitis optica spectrum disorder (NMOSD) is a relapsing inflammatory central nervous system disorder mainly involving the optic nerve and spinal cord. Except for the high recurrence rate and disability rate [1, 2], pain is highly prevalent in patients with NMOSD [3–5]. Pain has been an important factor affecting patients’ quality of life [6–10]. Neuropathic pain (NP) is one of types of pain being most characteristic [3, 4, 6]. However, there was no satisfactory treatment so far. At the same time, several studies have found that high doses of painkillers do not cure pain, but are associated with more severe cognitive impairment and fatigue [4, 11].

Several clinical observations suggested that some factors such age, myelitis and mood were associated with NP in patients with NMOSD [3, 12, 13]. Our study was designed to investigate the risk factors of NP among NMOSD patients.

## Methods

### Patients

**Fig. 1:**
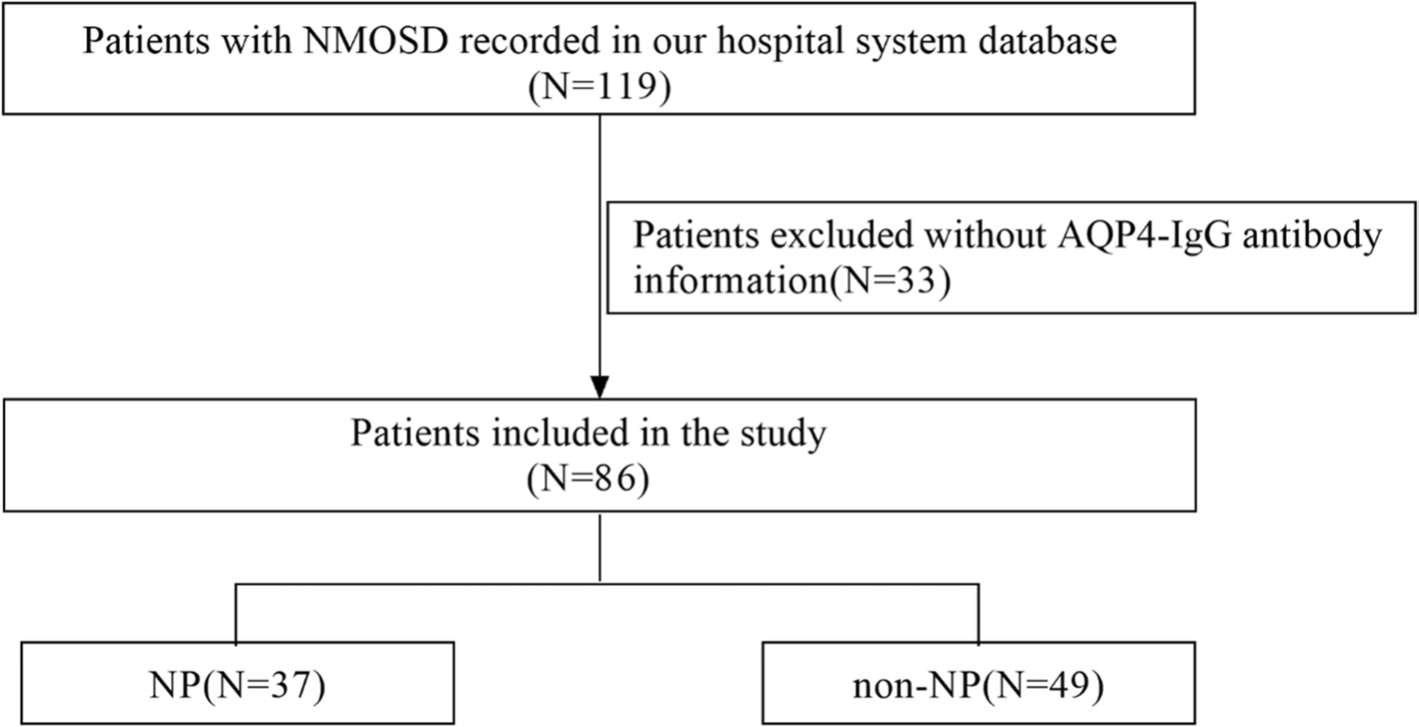
Flowchart depicting patients included in this study. NMOSD = neuromyelitis optica spectrum disorder, AQP4-IgG = aquaporin-4 immunoglobulin G, NP = neuropathic pain

Our study was a retrospective case-cohort study, the patients diagnosed with NMOSD in the Department of Neurology from the Second Affiliated Hospital of Guangzhou University of Chinese Medicine from January 2011 to October 2021 were screened. Inclusion criteria were (1) patients diagnosed as NMOSD according to the International Panel for NMO Diagnosis (IPND) criteria [14], (2) the aquaporin-4 immunoglobulin G antibodies (AQP4-IgG) test was performed (Fig. 1) [15]. Patients without AQP4-IgG antibody were excluded. Clinical data, including sex, age of the first onset, symptoms of the first onset including neuropathic pain and attack types, localization of lesions of the first onset on Magnetic Resonance Imaging (MRI), Extended disability status Scale (EDSS) of the first onset, treatment of immunosuppression in the first acute phase, disease modifying therapy (DMT), treatment of neuropathic pain and APQ4-IgG status were collected from the hospital system database. Neuropathic pain was defined according to the International Association for the Study of Pain criteria and was described as “pain arising as a direct consequence of a lesion or disease affecting the somatosensory system” [16]. The patients were divided into NP group and non-NP group. This study was approved by the Ethics Committee of the Second Affiliated Hospital of Guangzhou University of Chinese Medicine (YE2021-308).

### Statistical analyses

The dichotomous data were reported as the number with percentage, and the continuous data were expressed as the mean and standard deviation. The categorical variables were analyzed with a chi-square test. The continuous variables with normal distribution were analyzed using an independent two-sample Student’s t-test and data that were not normally distributed were analyzed with a nonparametric test. The variables that were statistically significant in the univariate analysis at *P* value less than 0.1 were included in a multivariate logistic regression model to assess the independent risk factors of neuropathic pain among NMOSD patients. The cut-off for statistical significance was *P* value less than 0.05.

## Result

### Demographic and clinical characteristics

Totally, 119 patients with NMOSD were screened. After the exclusion of 33 patients without AQP4-IgG antibody information, 86 patients were enrolled in this study (Fig. 1). Among the 86 patients, 75 (87.2%) patients were women, mean age of the first onset was 46.2 years. 74 (86.0%) patients with NMOSD were positive for the AQP4-IgG antibody. At the first attack, the mean EDSS is 4.3 ± 2.7 in NMOSD patients. The predominant clinical presentation was myelitis (73.3%) followed by optic neuritis (31.4%). 8 (11.9%) patients had optic lesions, 18 (26.9%) patients had brain stem lesions and 43 (50.0%) patients had spinal cord lesions. The predominant spinal lesion type was thoracic lesion (49.3%) followed by cervical lesion (44.8%). 27 (40.3%) patients had upper thoracic lesions (thoracic 1-thoracic 6). 18 (26.9%) patients had lower thoracic lesions (thoracic 7-thoracic 12). 18 (26.9%) patients had extended thoracic lesions (≥ 4 thoracic lesions). 40 (59.7%) patients had extended spinal cord lesions (≥ 3 spinal lesions). Intravenous methylprednisolone (87.8%) and intravenous immunoglobulin (21.6%) were used mainly in the acute phase, and oral glucocorticoid (96.5%), azathioprine (52.3%), and mycophenolate mofetil (15.1%) were used mainly in the remission phase (Table 1).

**Table 1:** Basic clinical characteristics of 86 patients with NMOSD

Characteristic	NMOSD(*n* = 86)
Female: male (ratio)	75:11(7:1)
Age, y, mean ± sd	46.2 ± 13.8
AQP4-IgG-positive, n (%)	74(86.0)
History of myelitis during the disease, n (%)	83(96.5)
EDSS, mean ± sd	4.3 ± 2.7
Length of spinal cord involvement, mean ± sd^a^	4.4 ± 4.7
Attack type, n (%)	
Optic neuritis	27(31.4)
Myelitis	63(73.3)
Brain stem	17(19.8)
MR lesion type, n (%)^a^	
Cervical	30(44.8)
Thoracic	33(49.3)
Lumber	2(3.0)
Brain stem	18(26.9)
Optic	8(11.9)
Isolated thoracic, n (%)^a^	13(19.4)
Upper thoracic lesions, n (%)^a^	27(40.3)
Lower thoracic lesions, n (%)^a^	18(26.9)
≥ 4 thoracic lesions, n (%)^a^	18(26.9)
≥ 3 spinal lesions, n (%)^a^	40(59.7)
IVMP, n (%)	65(87.8)
Intravenous immunoglobulin, n (%)	16(21.6)
Plasma exchange, n (%)	2(2.7)
Oral glucocorticoid, n (%)	83(96.5)
AZA, n (%)	45(52.3)
MMF, n (%)	13(15.1)

*AQP4-IgG* Aquaporin-4 immunoglobulin G, *EDSS* Extended disability status Scale, *Th* Thoracic, *Th1-Th6* Upper thoracic lesions, *Th7-Th12* lower thoracic lesions, *IVMP* Intravenous methylprednisolone, *AZA* Azathioprine, *MMF* Mycophenolate mofetil

^a^ Missing 19 cases

### Univariate analysis

In our study, 37 (43.0%) patients had neuropathic pain. The mean age of the first onset in the NP group was higher than in the non-NP group (51.2 ± 13.3 years VS 42.5 ± 13.2 years) (OR 1.05 [1.02–1.09], *P* = 0.006). The mean EDSS of patients in NP and non-NP group was separately 4.5 ± 2.6 and 4.0 ± 2.8 (*P* = 0.435). The mean length of spinal cord involvement of patients with NP was longer than patients in non-NP group (5.8 ± 5.0 VS 3.0 ± 3.8) (OR 1.16 [1.03–1.32], *P* = 0.018). 32 (86.5%) patients with NP had myelitis while only 31(63.3%) patients in non-NP group had myelitis during the first episode of the disease (OR 3.72 [1.23–11.24], *P* = 0.02). 7(18.9%) and 20(40.8%) patients in NP group and non-NP group had optic neuritis (OR 0.34 [0.12–0.92], *P* = 0.034). About the localization of the lesions, the percentage of the patients with cervical lesions was similar between two groups. There were more patients with thoracic lesion in NP group (61.8%) than non-NP group (36.4%). 1 (2.9%) patient in NP group and 7 patients (21.2%) in non-NP group had optic lesions (*P* = 0.047). The proportion of patients with upper thoracic lesions in the NP group was higher than in the non-NP group (52.9% VS 27.3%) (OR 3 [1.08–8.32], *P* = 0.035). The percentage of patients with lower thoracic lesions in the NP group was higher than in the non-NP group (38.2% VS 15.2%) (OR 3.47 [1.07–11.24], *P* = 0.038). The proportion of patients with extended thoracic lesions (≥ 4 thoracic lesions) in the NP group was higher than in the non-NP group (44.1% VS 9.1%) (OR 7.9 [2.01–30.95], *P* = 0.003). Among the patients with myelitis lesions, the proportion of patients with extended spinal cord lesions (≥ 3 spinal cord lesions) in the NP group was higher than in the non-NP group (76.5% VS 42.4%) (OR 4.41 [1.54–12.62], *P* = 0.006). Among them, more patients with neuropathic pain use intravenous immunoglobulin in the acute phase (35.3% VS 10%,*P* = 0.013), and more patients with neuropathic pain used mycophenolate mofetil during remission (24.3% VS 8.2%, *P* = 0.047). In terms of pain treatment, 13.5% of patients with neuropathic pain did not use analgesics, 86.5% used antiepileptic drugs, and 24.3% used antidepressants (Table 2).

**Table 2:** The univariate analysis of neuropathic pain among patients with NMOSD

Characteristic	NP (*n* = 37)	Non-NP(*n* = 49)	OR (95% CI)	*P* value
Female: male (ratio)	33:3(11:1)	42:8 (5:1)	2.21(0.54–8.99)	0.267
Age, y, mean ± sd	51.2 ± 13.3	42.5 ± 13.2	1.05(1.02–1.09)	0.006
AQP4-IgG-positive, n (%)	34(91.1)	40(81.6)	2.55(0.64–10.18)	0.174
Length of spinal cord involvement, mean ± sd^a^	5.8 ± 5.0	3.0 ± 3.8	1.16(1.03–1.32)	0.018
EDSS, mean ± sd	4.5 ± 2.6	4.0 ± 2.8	1.07(0.9–1.28)	0.435
Attack type, n (%)				
Optic neuritis	7(18.9)	20(40.8)	0.34(0.12–0.92)	0.034
Myelitis	32(86.5)	31(63.3)	3.72(1.23–11.24)	0.02
Brain stem	9(24.3)	8(16.3)	1.65(0.57–4.79)	0.359
MR lesion type, n (%)^a^				
Cervical	19(55.9)	11(33.3)	2.53(0.94–6.83)	0.066
Thoracic	21(61.8)	12(36.4)	2.83(1.05–7.61)	0.04
Lumber	1(2.9)	1(3.0)	0.97(0.06–16.17)	0.983
Brain stem	10(29.4)	8(24.2)	1.3(0.44–3.86)	0.634
Optic	1(2.9)	7(21.2)	0.11(0.01–0.97)	0.047
Isolated thoracic, n (%)^a^	8(23.5)	5(15.2)	1.72(0.5–5.94)	0.389
Upper thoracic lesions, n (%)^a^	18(52.9)	9(27.3)	3(1.08–8.32)	0.035
Lower thoracic lesions, n (%)^a^	13(38.2)	5(15.2)	3.47(1.07–11.24)	0.038
≥ 4 thoracic lesions, n (%)^a^	15(44.1)	3(9.1)	7.9(2.01–30.95)	0.003
≥ 3 spinal lesions, n (%)^a^	26(76.5)	14(42.4)	4.41(1.54–12.62)	0.006
IVMP, n (%)	32(94.1)	33(82.5)	3.39(0.66–17.58)	0.145
Intravenous immunoglobulin, n (%)	12(35.3)	4(10)	4.91(1.41–17.13)	0.013
Plasma exchange, n (%)	0(0)	2(5)	0(0–0)	0.999
Oral glucocorticoid, n (%)	37(100)	46(93.9)	Inf	0.999
AZA, n (%)	19(51.4)	26(53.1)	0.93(0.4–2.19)	0.875
MMF, n (%)	9(24.3)	4(8.2)	3.62(1.02–12.86)	0.047
Antidepressants, n (%)	9(24.3)	-	-	-
Antiepileptic, n (%)	32(86.5)	-	-	-
Antispasticity, n (%)	12(32.4)	-	-	-
Opioids, n (%)	2(5.4)	-	-	-

*AQP4-IgG* Aquaporin-4 immunoglobulin G, *EDSS* Extended disability status Scale, *Th1-Th6* Upper thoracic lesions, *Th7-Th12* Lower thoracic lesions, *NP* Neuropathic pain, *IVMP* Intravenous methylprednisolone, *AZA* Azathioprine, *MMF* Mycophenolate mofetil

^a^ Missing 19 cases

### Multivariate analysis

In multivariate regression analysis, extended thoracic lesions (≥ 4 thoracic lesions) were independent risk factors of neuropathic pain in NMOSD patients (OR 20.21 [1.18–346.05], *P* = 0.038) after adjusting for age, gender, optic neuritis, upper thoracic lesions, intravenous immunoglobulin, MMF and EDSS. Meanwhile, age was an independent risk factor of neuropathic pain in NMOSD patients (OR 1.35 (1–1.81) *P* = 0.050). Gender might be also an independent risk factor of neuropathic pain in NMOSD patients (OR 12.11 (0.97–151.64) *P* = 0.053) (Table 3).

**Table 3:** The multivariate analysis of neuropathic pain among patients with NMOSD. (neuropathic pain as dependent variable)

	Crude		Adjusted model	
	OR (95% CI)	*P* value	OR (95% CI)	*P* value
Age at the onset, each 5 years	1.30(1.08–1.56)	0.006	1.35(1–1.81)	0.05
Gender, female	2.21(0.54–8.99)	0.267	12.11(0.97–151.64)	0.053
Optic neuritis	0.34(0.12–0.92)	0.034	0.43(0.05–3.66)	0.443
Upper thoracic lesions^a^	3.00(1.08–8.32)	0.035	0.75(0.11–4.97)	0.763
≥ 4 thoracic lesions^a^	7.90(2.01–30.95)	0.003	20.21(1.18–346.05)	0.038
EDSS	1.07(0.90–1.28)	0.435	1.19(0.91–1.56)	0.216
Intravenous immunoglobulin	4.91(1.41–17.13)	0.013	2.43(0.5–11.73)	0.269
MMF	3.62(1.02–12.86)	0.047	7.32(0.83–64.15)	0.072

Adjusted for age at the onset, gender, optic neuritis, upper thoracic lesions, ≥ 4 thoracic lesions, EDSS, Intravenous immunoglobulin, MMF

*AQP4-IgG* Aquaporin-4 immunoglobulin G, *Th* Thoracic, *Th1-Th6* Upper thoracic lesions, *EDSS* Extended disability status Scale, *MMF* Mycophenolate mofetil

^a^ Missing 19 cases

## Discussion

The key finding of the present study was that among NMOSD patients, extended thoracic lesions (≥ 4 thoracic lesions) were independent risk factors of neuropathic pain in NMOSD patients which were not reported in the previous studies. Patients with extended thoracic lesions had a 20.21-fold higher risk of neuropathic pain. Furthermore, age and gender might be also associated with neuropathic pain.

### Correlation between spinal lesion level and neuropathic pain

The prevalence of neuropathic pain in patients with NMOSD was 31.5-85.5% [3, 6, 12, 17]. Consistent with the previous studies, the prevalence of neuropathic pain in our study was 43%.

About the mechanism of neuropathic pain, the possible hypothesis was that neurons with bilateral descending projections to the lumbosacral superficial dorsal horn were concentrated in the autonomic intermediomedial nucleus surrounding the central canal of the upper/mid-thoracic spinal cord, and lesions of the upper/mid-thoracic segment may accompany pain easily [18].

On the basis of this hypothesis, some studies began to investigate the relationship between neuropathic pain of NMOSD patients and the spinal cord. Several studies found that the number of involved spinal segments (OR 1.14 [1.03–1.28], *P* = 0.024), especially the upper 6 thoracic segments (OR 1.31 [1.01–1.63], *P* = 0.018) was associated with pain among NMOSD patients, and that pain score was higher in patients with than in those without extended spinal cord lesions (≥ 3 spinal cord lesions) (median, 17.5 vs. 10.0; IQR, 9.5 vs. 10.0; *P* = 0.036) [3, 19]. In Oxford and Liverpool’s prospective study, they showed that the presence of thoracic lesions (std. β =  − 0.46, *P* = 0.03) predicts greater myelitis-associated chronic pain than the presence of cervical lesions (std. β = 0.48, *P* = 0.04) [12]. On the contrary, another cohort showed that there was no significant difference in the length of the lesion between NP and non-NP groups (8.8 VS 7.6 spinal cord segments, respectively, *P* = 0.422) [6].

However, these studies suggested the number of involved spinal segments especially the upper 6 thoracic segment or the presence of thoracic lesions or extended spinal cord lesions (≥ 3 spinal lesions) might be associated with pain among NMOSD patients while our study pointed out the extended thoracic lesions(≥ 4 thoracic cord lesions) was an independent risk factor of neuropathic pain among patients with NMOSD which was the novelty of the present paper. Since the association between neuropathic pain and the features of spinal lesions is still controversial, prospective cohort studies were needed.

### Correlation between gender and neuropathic pain

In this study, gender might be also an independent risk factor of neuropathic pain in NMOSD patients which was not found in the previous studies [17, 20]. Female patients had a 12.11-fold higher risk of neuropathic pain than male patients. Up to now, there is an unknown mechanism between gender and neuropathic pain in NMOSD patients. But several studies advanced the hypothesis that there is a difference between the sexes in the mechanism of the initiation and maintenance of neuropathic pain. Firstly, neuroinflammation which could cause pain is driven by different cells. Neuroinflammation seems to have more microglia involved in males, whereas in females it seems to be driven primarily by T lymphocytes. Secondly, sex hormones such as testosterone might alter the main immune cells that cause neuroinflammation in the nervous system, which in turn alters the pathways that cause pain [21–25]. Except for those, some women might have a genetic predisposition to chronic pain [26]. In conclusion, gender may influence the initiation and maintenance of neuropathic pain. However, the association of gender and neuropathic pain needs more basic researches and clinical researches to verify.

### Correlation between age and neuropathic pain

The correlation of neuropathic pain and age might be related to decreased small fibers and a declined neural plasticity [27, 28]. Previous study showed controversial results [12, 20]. In our study, the age of NMOSD patients with neuropathic pain was higher than those without in this study (51.2 ± 13.3 VS 42.5 ± 13.2, *P* = 0.006). The multivariate analysis showed that age might increase the incidence of NP among NMOSD patients (OR 1.35 (1–1.81) *P* = 0.050).

### Correlation between AQP4-IgG antibody status and neuropathic pain

Until now, there were several hypotheses about the mechanism of neuropathic pain related with AQP4-IgG antibody status among NMOSD patients.

Firstly, under physiological conditions, AQP4 is co-expressed with the excitatory amino acid transporter 2, which enables glutamate uptake by astrocytes [29]. Glutamine is the material basis of γ-aminobutyric acid (GABA) [30]. Loss of AQP4 may lead to an excessive accumulation of glutamate in the extracellular space and reduce the production of GABA.

Secondly, extensive loss of AQP4 could induce reactive astrocytes. A2 astrocytes secrete neuroprotective factors that promote neuronal survival, and then may inhibit the progression of chronic pain. A1 astrocytes not only secrete toxic factors that rapidly kill mature oligodendrocytes and neurons but also produce small molecules such as cytokines, chemokines and growth factors that influence the glutamine-glutamate-GABA axis [31–33].

Therefore, the reactive astrocyte played an important role in the development of neuropathic pain among NMOSD patients.

Some clinical studies found that patients had neuropathic pain were tended to be AQP4-IgG-positive than those without pain (89.55% VS 79.07%, *P* = 0.082) [34]. In our study, the proportion of AQP4-IgG-positive patients in the NP group was higher than in the non-NP group (91.1% VS 81.6%, *P* = 0.174). Whether the neuropathic pain was associated with AQP4-IgG antibody status needed more studies to verify.

### Limitation

As a retrospective study, the pain score of patients at the onset was not assessed. Therefore, we could not find out whether extended thoracic lesions were associated with the severeness of neuropathic pain. Further prospective studies with a more detailed evaluation of neuropathic pain were needed to verify our conclusion.

## Conclusions

Extended thoracic lesions (≥ 4 thoracic lesions), age and gender might be independent risk factors of neuropathic pain among patients with NMOSD. However, with a small sample size and predominantly female, caution must be applied and these results need validating in further cohorts.

## Data Availability

The datasets generated and/or analysed during the current study are not publicly available due privacy or ethical restrictions but are available from the corresponding author on reasonable request.

## Abbreviations

NMOSD: Neuromyelitis optica spectrum disorder
AQP4-IgG: Aquaporin-4 immunoglobulin G
NP: Neuropathic pain
GABA: γ-Aminobutyric acid
Th: Thoracic
MRI: Magnetic Resonance Imaging
IPND: International Panel for NMO Diagnosis
OR: Odds ratio
CI: Confidence interval
IVMP: Intravenous methylprednisolone
AZA: Azathioprine
MMF: Mycophenolate mofetil
EDSS: Extended disability status Scale
